# Electroencephalography power and coherence changes with age and motor skill development across the first half year of life

**DOI:** 10.1371/journal.pone.0190276

**Published:** 2018-01-12

**Authors:** Ran Xiao, Joanne Shida-Tokeshi, Douglas L. Vanderbilt, Beth A. Smith

**Affiliations:** 1 Division of Biokinesiology and Physical Therapy, University of Southern California, Los Angeles, California, United States of America; 2 Department of Pediatrics, Division of General Pediatrics, Keck School of Medicine, University of Southern California, Los Angeles, California, United States of America; University of Electronic Science and Technology of China, CHINA

## Abstract

Existing research in infants has correlated electroencephalography (EEG) measures of power and coherence to cognitive development and to locomotor experience, but only in infants older than 5 months of age. Our goal was to explore the relationship between EEG measures of power and coherence and motor skill development in younger infants who are developing reaching skill. Twenty-one infants with typical development between 38 and 203 days of age participated. Longitudinal EEG recording sessions were recorded in monthly increments, with 3–5 sessions acquired for 19 participants and 1 session for 2 participants, resulting in 71 sessions in total. EEG variables of interest were relative power in the 6–9 Hz range and coherence between selected electrode pairs. We describe the development of the peak in relative power in the 6–9 Hz frequency band of EEG; it is not present around 1 month of age and starts to appear across the following months. Coherence generally increased in the bilateral frontal-parietal networks, while the interhemispheric connectivity in motor cortices generally decreased. The results of this relatively small pilot study provide a foundational description of neural function changes observed as motor skills are changing across the first half year of life. This is a first step in understanding experience-dependent plasticity of the infant brain and has the potential to aid in the early detection of atypical brain development.

## Introduction

Electroencephalography (EEG) power represents amount of activity in certain frequency bands of the signal while coherence between different electrodes reflects the degree to which connections are present across brain regions [1]. Existing research in infants has correlated EEG measures of power and coherence to cognitive development and to locomotor experience, but only in infants older than 5 months of age. Changes in the power of the infant mu rhythm can be measured between 5 and 7 months of age [2], while alpha desynchronization prior to the initiation of an arm reach to grasp an offered toy can be observed in infants 9 months of age [3]. Researchers have demonstrated differences in the power and coherence of EEG signals of 8-month-old typically-developing infants with various amounts of crawling experience [4] and, in 12-month-old typically-developing infants with various amounts of walking experience [5]. Our goal in the present study was to complement previous studies demonstrating a relationship between infant mu rhythm development and motor skills, while expanding upon them by assessing a novel group: younger infants who are developing reaching skill.

As described in a recent review article, Gonzalez and colleagues [6] stated, “As reviewed here, research utilizing power, coherence, and mu desynchronization provides great insight on motor, cognitive, and social development as *isolated* domains…. Much work is left to be done, as little is known regarding the longitudinal development of measures like mu desynchronization, or how power, coherence, and mu desynchronization relate to our existing knowledge of motor, cognitive and social development.” We have addressed this gap by longitudinally measuring EEG power and coherence in the mu frequency band across the development of arm reaching skill in infants 1–7 months of age. This work provides fundamental insight into neural function changes observed as motor skills are changing, and has the potential to inform both our understanding of experience-dependent plasticity of the infant brain and to aid in the early detection of atypical brain development.

## Materials and methods

### Experiment design and data acquisition

This study was approved by the Institutional Review Board of the University of Southern California, and informed consent was obtained from a parent or legal guardian before participation. Twenty-one infants with typical development between 38 and 203 days of age participated in the present study. Infants were from singleton, full-term (38 weeks minimum gestation) births. Infants experiencing complications during birth, or with any known visual, orthopedic or neurologic impairment or a score at or below the 5^th^ percentile for their age on the Bayley Scales of Infant Development (3^rd^ edition) [7] at the time of testing were excluded. Longitudinal EEG recording sessions were recorded in monthly increments, with 3–5 sessions acquired for 19 participants and 1 session for 2 participants. That resulted in 71 sessions in total.

**Fig 1:**
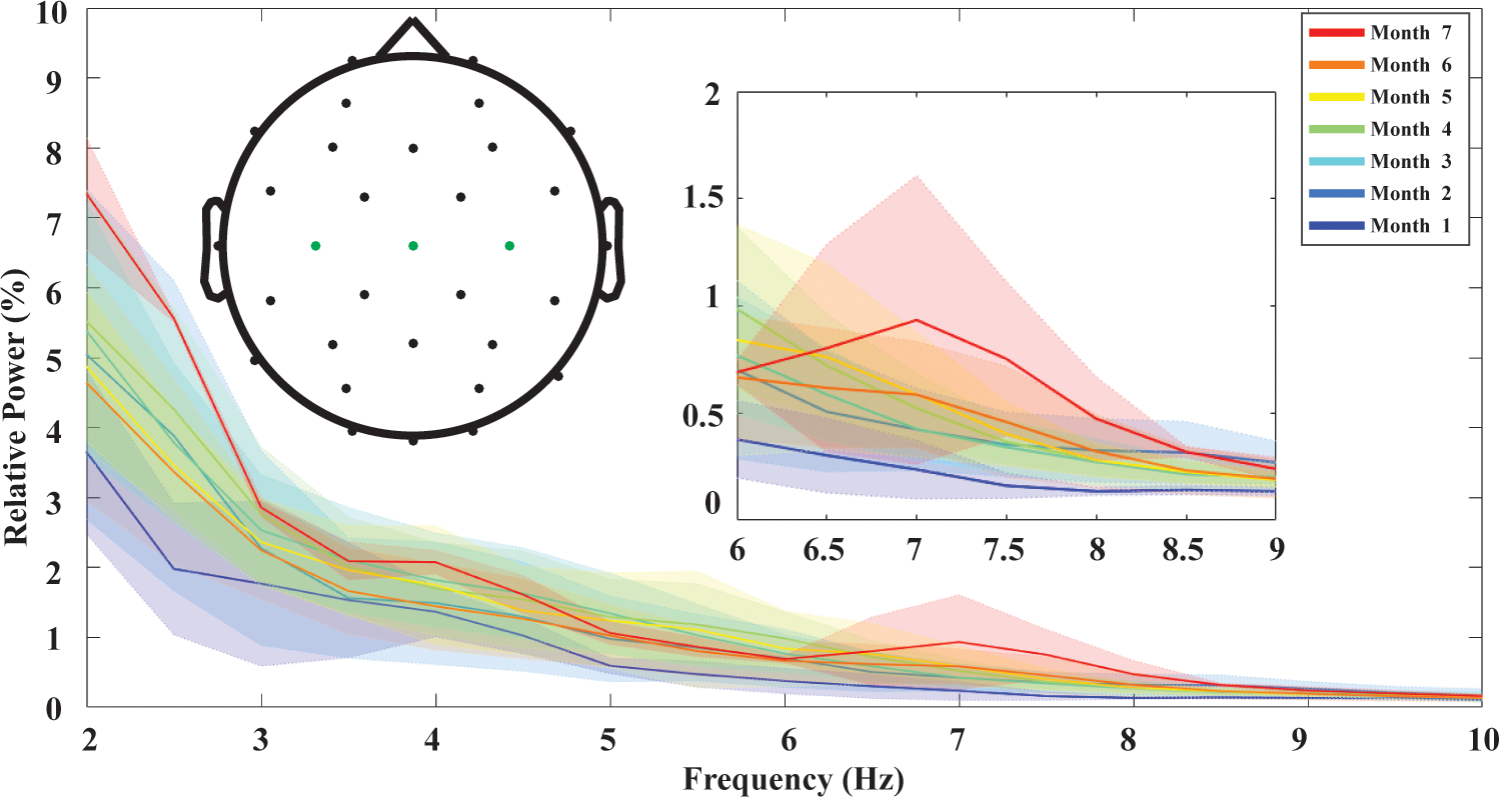
Monthly spectral profiles at motor cortices. Each color-coded curve presents averaged relative power density from sessions of the same monthly age. Shaded areas show standard deviations for each monthly age with corresponding colors. Left inset shows geodesic locations of each electrode on the scalp, and green ones are selected to evaluate spectral profiles in motor cortices. Right inset provides a zoom-in view of spectral profiles in 6–9 Hz.

During each experimental session, EEG data were noninvasively acquired using a Biosemi system with 32-electrode EEG caps (electrode locations are presented in Fig 1) at sampling rate of 512 Hz. Different sizes of EEG caps were utilized to accommodate infants of different head circumferences and head growth during the study. For EEG recordings, trials were designed in the following fashion. First, 2 trials of 20-second baseline EEG data were recorded. Experimenters were encouraged to push baseline recordings to 1 minute if participants were calm and cooperative. During baseline recording, a glowing globe toy was presented out of participants’ reaching distance to attract their attention and minimize body movements. Second, a toy that each participant was interested in was presented in front of them, within reach at midline, encouraging them to reach in 20-second duration. This served as the reaching condition. Third, the toy was removed and corresponding EEG was recorded for 20 seconds as a non-reaching condition. The reaching and non-reaching trials were alternatively repeated for 5 times. Last, the same baseline condition as the first one was recorded one more time. The present study is an integral part of an on-going project in a larger scope to investigate neuromotor development during infancy, and only the baseline parts of EEG recordings were included in the analysis in this study. Video data were recorded at the same time using a digital camera, with which a reaching skill score was derived. Specifically, an experimenter carefully examined the video of each session after experiment and assigned one of four reaching skill levels 1) none = the infant does not move the arm toward the toy, 2) low = the infant moves the arm toward the toy, may touch it but does not grasp it, 3) medium = the infant moves the arm toward the toy, path may be indirect, toy grasped without pre-shaped hand, 4) high = the infant moves the arm directly toward the toy and grasps it with a pre-shaped hand. Bayley Scales of Infant Development (3^rd^ edition) [7] was administered to measure cognitive, language and motor development. Wearable sensor data from full-day arm movement and anthropometric data were also recorded but will not be discussed further here.

### Preprocessing of EEG

Comparing to invasive EEG, noninvasive EEG recordings impose less than minimal risk for participants, but are relatively low in signal-to-noise ratio and very susceptive to external noises. Hence a series of preprocessing techniques were adopted to enhance the signal quality. First, EEG data from all electrodes were re-referenced to the average of T7 and T8 (electrodes at left and right temporal lobes close to ears). By default, the Biosemi system records EEG by referencing each electrode to a common mode sense (CMS) active electrode, and common mode information exists in the recorded signals. This re-reference step helps achieve the system’s full 80 dB common-mode rejection ratio (CMRR). Second, a bandpass infinite impulse response filter (0.3–30 Hz) was applied to the re-referenced data to remove direct current offset and interference from high frequency components (powerline noise, etc.). The frequency range was selected to contain infant alpha activities within 6–9 Hz, while minimizing effects from other sources. Third, baseline conditions from EEG recordings were extracted and segments with very large fluctuations were visually identified and removed from further analysis. Fourth, Kurtosis indices were calculated for all electrodes. Electrodes with Kurtosis indices falling beyond 5 standard deviations of all electrodes were marked and rejected. Their data were then interpolated by the average of surrounding electrodes. Fifth, a common average reference was applied by re-referencing each electrode to the average of all electrodes in order to spatially filter out common-mode artifacts. Last, an independent component analysis was performed to decompose baseline EEG into independent components (IC) from brain sources and unwanted artifacts [8]. Components caused by electrocardiography, lateral eye movements, eye blinking, motion artifacts, etc. were visually identified and removed to further improve signal quality for the following analysis. To maximally preserve information regarding brain activities, each IC was carefully examined by the combination of its temporal, spectral and spatial patterns, resulting in 2 or 3 ICs being rejected for most sessions. The preprocessing steps were performed using EEGLAB toolbox (ver. 13_6_5_b) [9].

### Spectral analysis

To capture age related changes in alpha activities in infant EEG, power spectral density (PSD) was estimated on the preprocessed EEG data using Welch’s method [10]. A Hann window with window length of 2 seconds was chosen for the PSD estimation, with 50% overlap between segments. This resulted in a frequency resolution of 0.5 Hz to capture changes in spectral activities in infant EEG. The “pwelch” function in MATLAB (ver. 2016A, MathWorks Inc., Natick, MA, USA) was implemented for the PSD estimation.

Due to variation across sessions and ages, PSDs were transformed into relative powers so that spectral activities from all individual sessions were set to an equal footing for comparison. The relative powers were calculated between 0 and 30 Hz. For each frequency bin within this range and each electrode, relative power was computed by dividing PSD by the sum PSD from all bins. Such transformation adjusted the original PSDs into the energy ratios within sub 30 Hz frequency range, and fair cross-session comparison could be achieved. Alpha band powers were then computed for each session by adding up relative powers of all frequency bins within 6–9 Hz, and alpha activities from motor cortices were achieved by averaging key representative electrodes from left, medial and right motor cortices (C3, Cz and C4, as shown in left inset in Fig 1).

The changes in alpha activities at early life were assessed from three aspects. Sessions with the same monthly ages were grouped and their alpha activities from motor cortices were averaged to provide a qualitative view of changes in 6–9 Hz activities along maturation. Then alpha band powers from each electrode were registered to their geodesic locations on the scalp to provide information about developmental changes in spatial domain. Aside from changes in spectral profiles and spatial patterns, linear relationship between alpha activities in motor cortices from each session and their corresponding chronological age in days was investigated by calculating their Pearson correlation. This provided a quantitative evaluation of changes in alpha activities along maturation, and a least-squares fitted line was also constructed to present the changing trend.

### Connectivity analysis

The magnitude-squared coherences between electrode pairs were computed to study changes in alpha activities across different brain regions. Welch’s method was implemented to estimate PSD for each electrode of the interested electrode pair as well as cross-electrode PSD [10]. Then magnitude-squared coherence was calculated by

$$Coh_{E_{1}E_{2}}\left(f\right)=\frac{{\left|PSD_{E_{1}E_{2}}\left(f\right)\right|}^{2}}{PSD_{E_{1}}\left(f\right)•PSD_{E_{2}}\left(f\right)}$$ (1)

where $Coh_{E_{1}E_{2}}\left(f\right)$ was the magnitude-squared coherence between electrode *E*_1_ and *E*_2_ at frequency *f*; $PSD_{E_{1}}$ and $PSD_{E_{2}}$ were their corresponding PSD estimations, and $PSD_{E_{1}E_{2}}$ was the cross-electrode PSD estimation. Again, Hann window with length of 2 seconds and 50% overlap was chosen in the PSD estimation, to be consistent with previous spectral analysis. The MATLAB function “mscohere” was adopted for the coherence calculation. To compare changes across different sessions, mean alpha coherence was defined as the average of coherences across all bins within 6–9 Hz. In total, 18 pairs of electrodes were selected to investigate changes in connectivity across different functional brain regions, including frontal, motor, and parietal cortices. A full list of electrode pairs could be found in Table 1.

**Table 1:** Likelihood ratio test of changes in mean alpha coherence across chronological age in days, reaching skill, fine motor and gross motor scores, for all 18 electrode pairs. A cutoff value of 0.05/18 (adjusted by number of electrode pairs) is selected for the statistical tests and p-values smaller than it are marked in bold and italic.

Electrode Pair	Chronological age (days)		Reaching scores		Fine motor scores		Gross motor scores	
	χ^2^	p	χ^2^	p	χ^2^	p	χ^2^	p
**Fz-Cz**	3.899	0.048	4.527	0.033	4.785	0.029	6.730	0.009
**Cz-Pz**	0.166	0.683	1.024	0.312	0.355	0.551	0.530	0.467
**Fz-Pz**	2.131	0.144	0.426	0.514	2.289	0.130	1.450	0.228
**F3-F7**	0.014	0.905	0.836	0.360	0.025	0.874	0.486	0.486
**F4-F8**	4.255	0.039	4.124	0.042	8.895	0.003	4.192	0.041
**F3-P3**	4.875	0.027	9.276	***0*.*002***	4.636	0.031	2.049	0.152
**F4-P4**	13.673	***0*.*000***	11.821	***0*.*001***	12.992	***0*.*000***	10.545	***0*.*001***
**F7-P3**	1.983	0.159	2.433	0.119	2.640	0.104	0.085	0.770
**F8-P4**	0.373	0.541	2.071	0.150	1.090	0.297	0.023	0.878
**F3-F4**	3.981	0.046	1.722	0.189	3.667	0.056	1.523	0.217
**F7-F8**	0.041	0.840	0.207	0.649	0.000	0.991	0.220	0.639
**C3-C4**	15.604	***0*.*000***	7.414	0.006	8.669	0.003	10.386	***0*.*001***
**P3-P4**	0.345	0.557	0.620	0.431	0.587	0.444	1.732	0.188
**P7-P8**	2.168	0.141	0.169	0.681	1.106	0.293	2.203	0.138
**F3-C3**	5.117	0.024	6.536	0.011	5.827	0.016	2.624	0.105
**F4-C4**	1.208	0.272	0.715	0.398	0.863	0.353	1.719	0.190
**C3-P3**	0.028	0.868	0.001	0.973	0.028	0.868	0.368	0.544
**C4-P4**	0.336	0.562	0.002	0.964	0.773	0.379	0.001	0.972

Developmental changes in brain connectivity were evaluated through studying changes in mean alpha coherences from each of these 18 electrode pairs along maturation and across key motor development scores (reaching skill score and Bayley Scales fine motor and gross motor subscale scores). Since there were multiple samples from most participants at different age points, the existence of repeated measurements made modeling with simple linear regression less effective. Alternatively, the linear mixed-effects model (LMM) was adopted in present study to model the relationship between mean alpha coherence and various predictors. Comparing to linear model, the LMM tackled the non-independency within the grouping factor (participants) by adding random effect terms to the model so that more accurate representation of outcomes could be achieved [11]. Specific LMM models were designed for the four types of predictors, due to their intrinsic characteristics, especially between age and key motor development scores, as following:

Coh∼Age+(1|Participant)+ε (2)

Coh∼Reaching+(1|Participant)+ε (3)

Coh∼FMS+(1|Participant)+ε (4)

Coh∼GMS+(1|Participant)+ε (5)

Eqs (2–5) presents LMM models designed for capturing changes in mean alpha coherences across age, reaching skill score, fine motor score and gross motor score, respectively. In Eq (2), *Coh* denotes mean alpha coherence; *Age* denotes chronological age in days during each session; *ε* is the error term. The term (1|*Participant*) refers to random intercepts within the grouping factor (participants) to alleviate effect from repeated measurements. For models with key motor development scores (*Reaching* as reaching skill score, *FMS* as Bayley fine motor score and *GMS* as Bayley gross motor score), grouping factors were also taken into consideration by adding the random intercept terms.

To test whether these predictors present significant effect on the changes of mean alpha coherence, likelihood ratio tests were conducted by firstly removing each targeted predictor from original models as null models and then comparing their goodness of fit to the original ones in Eqs (2–5). Likelihood ratio test was also performed on alpha band powers along maturation. In null model, random intercept was omitted in comparison to added random intercept term in the alternative LMM model to test the effect of repeated measures from different subjects. In all tests, the significance level was set at 0.05, and it is adjusted using the Bonferroni correction for multiple comparisons to account for the number of electrode pairs being evaluated (i.e., 0.05/18). For age, fine and gross motor scores, scatter plots with least-squares fitted lines were generated to present the changing trends and correlation coefficients were calculated to quantify their linear relationship with mean alpha coherence for each electrode pair. On the other hand, bar plots with mean and standard deviation were chosen to present changes in mean alpha coherence across different reaching skill scores, due to the sparse sample points.

## Results

### Spectral profiles and spatial patterns of alpha activities

Fig 1 presents the spectral profiles in 2–10 Hz of infant EEG at motor cortices from 1 to 7 months of age. Each solid curve represents spectral profile from one monthly age, color coded from cool to warm as age increasing. The shaded area shows the region within one standard deviation of sessions of the same monthly age. Large changes can be observed within 2–3 Hz, 3–5 Hz and 6–9 Hz bands. The former two do not present conclusive changing patterns along maturation, with monthly spectral profiles interleaving among earlier and later months of age. On the contrary, alpha activities from motor cortices present a quite consistent increasing pattern along maturation. The flat pattern for the spectral profile from 1 month of age gradually grows into an obvious bump shape at 7 months of age, as shown in the right inset of Fig 1.

**Fig 2:**
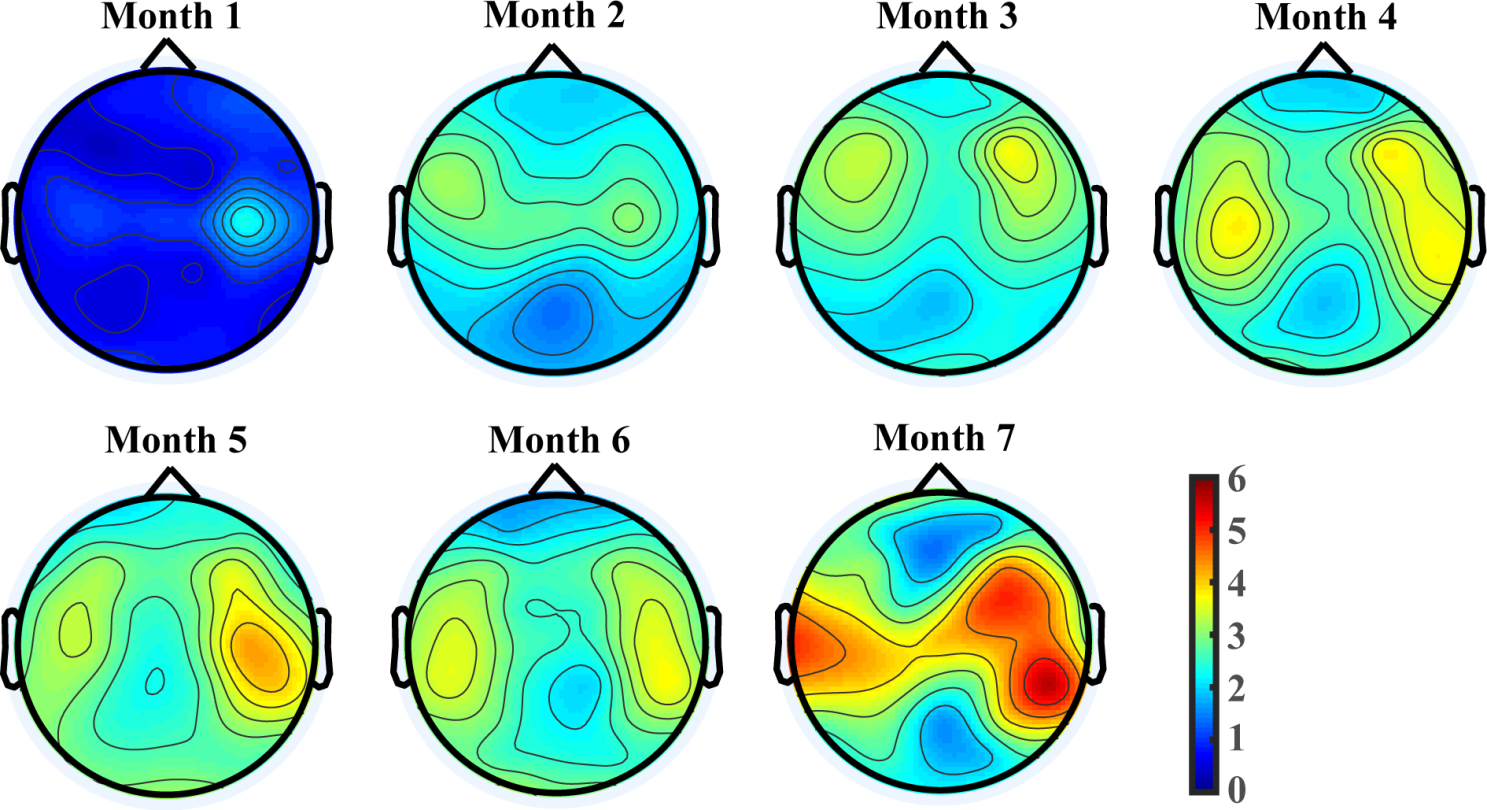
Topographies of alpha band power for each monthly age. First row shows spatial patterns of alpha band power from 1 to 4 months of age. Second row shows those for 5–7 months of age.

Fig 2 provides a topographic view of changes in alpha activities along chronological age. It shows alpha activities are most prominent in central regions covering motor cortices during the first 7 months of age comparing to other cortical regions. Even starting from 1 month of age, alpha activities cumulate at motor regions and present a spreading trend along maturation. Furthermore, it presents a generally increasing pattern in magnitude along maturation at the alpha dominated regions.

### Changes in alpha band powers along maturation

**Fig 3:**
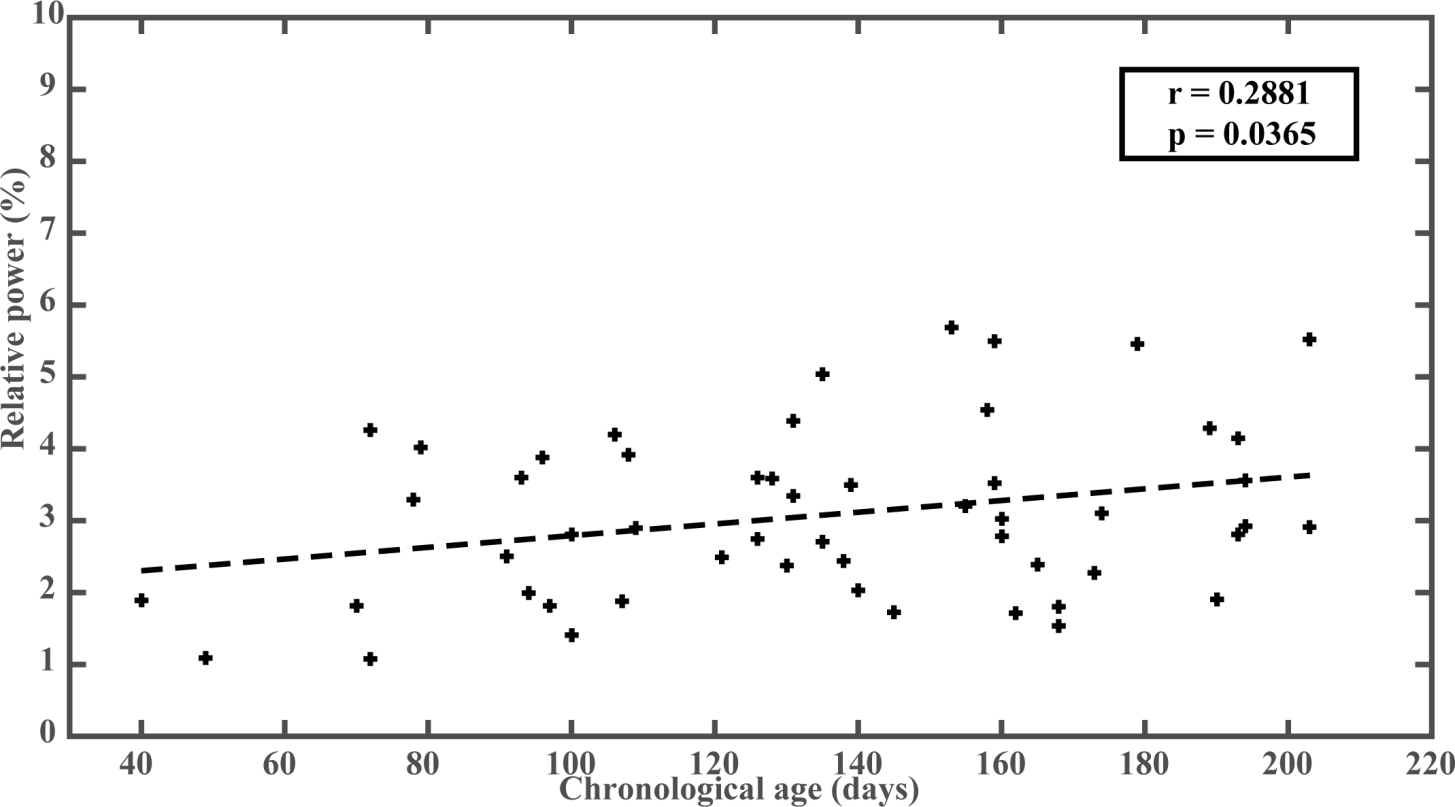
Correlation of alpha band power with chronological age in days. Each cross represents magnitude of alpha band power from one session. Dashed line shows the least-squares fitted line along maturation. The r and p values denote correlation test results.

Fig 3 provides quantitative evaluation of changes in alpha activities along maturation. Individual-session alpha band powers present an increasing linear trend, as indicated by the scatter plot and the least-squares fitted line. Correlation test confirms the trend with correlation coefficient of 0.2881 between alpha band powers at each session and their corresponding chronological age in days (*p < 0*.*05*). Due to the existence of repeated measurements, likelihood ratio test was conducted by constructing two LMM models with and without subject variation as random effect. The test demonstrates no significant impact from subject variation on the changes of alpha band powers along maturation (χ^2^ = 0.928, *p = 0*.*335*). We conclude that accounting for within-subjects effects does not change the interpretation of the relationship between increasing age and increasing alpha band power.

### Cortical reorganization along maturation

**Fig 4:**
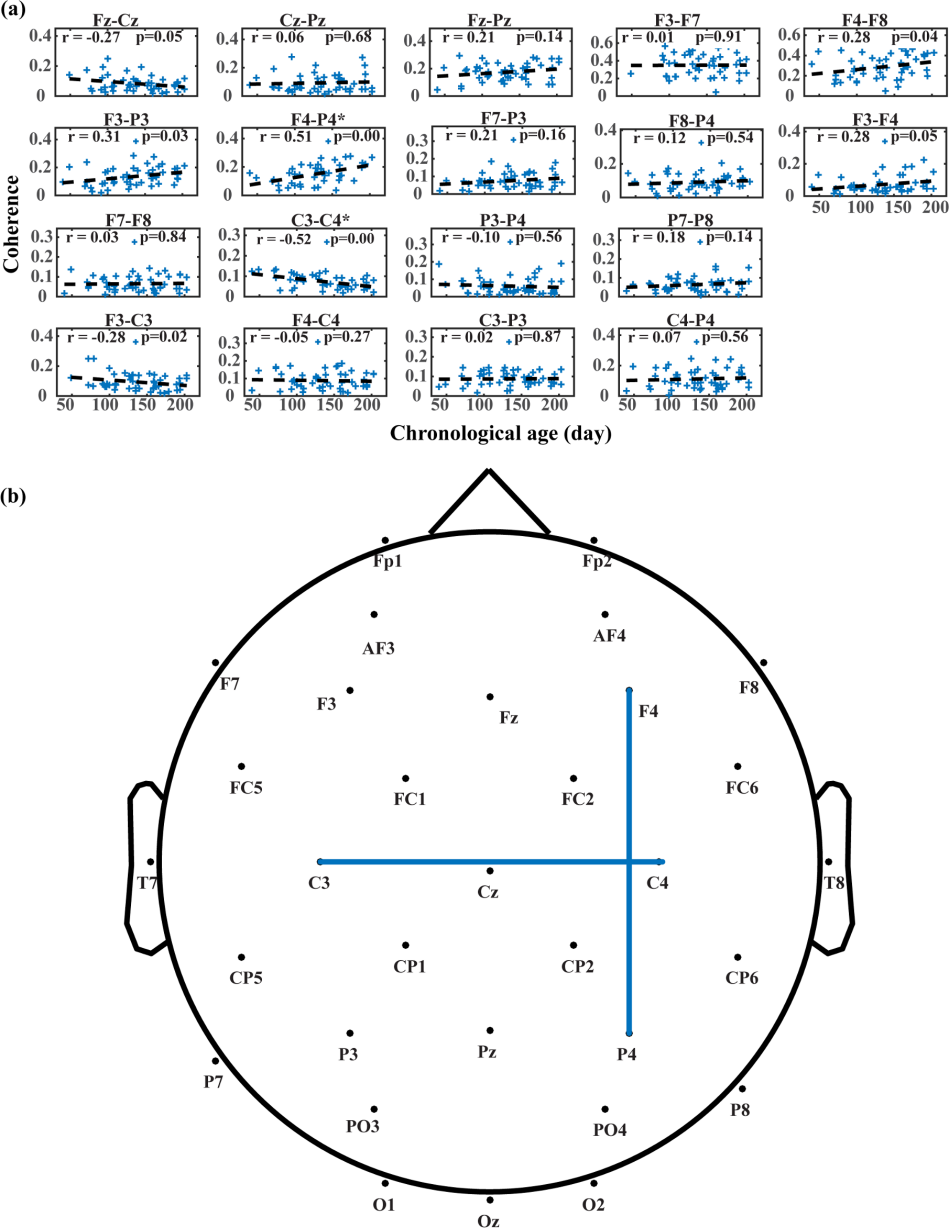
Changes in mean alpha coherence along maturation. (A) Changing trends of mean alpha coherence for each electrode pair. In each subplot, blue crosses denote magnitudes of mean alpha coherence for all sessions, and dashed lines are the least-squares fitted lines. (B) Electrode pairs with significance in likelihood ratio tests. Electrode pairs with significance are connected with blue lines.

Fig 4 presents changes in mean alpha coherence along chronological age in days. Fig 4A reveals distinct age-related changing trends of connectivity across different brain regions. Connections between some regions strengthen as infants grow, while others present a weakening pattern. Fig 4B reveals brain regions with significant changes of connectivity along maturation. Out of 18 electrode pairs evaluated in present study, 2 pairs present significant changes based on the Bonferroni corrected significance level, between right frontal (F4) and parietal (P4) (χ^2^ = 13.673, *p < 0*.*0028*), and left (C3) and right motor (C4) (χ^2^ = 15.604, *p < 0*.*0028*). Several other electrode pairs also present large changes, including left frontal (F3) and parietal (P3) (χ^2^ = 4.875, *p < 0*.*05*), left frontal (F3) and motor (C3) (χ^2^ = 5.117, *p < 0*.*05*), medial frontal (Fz) and motor (Cz) (χ^2^ = 3.899, *p < 0*.*05*), and right frontal (F4) and pre-frontal (F8) (χ^2^ = 4.255, *p < 0*.*05*) cortices. Frontal-motor connectivity presents a generally decreasing pattern, as shown in F3-C3 (r = -0.28) and Fz-Cz (r = -0.27) electrode pairs. For both sides of cortices, the frontal-parietal network strengthens in connection along maturation, as indicated by F3-P3 (r = 0.31) and F4-P4 (r = 0.51) electrode pairs. Within motor cortices, interhemispheric connection decreases when infants get older, with C3-C4 electrode pair presenting a clear negative correlation (r = -0.52).

### Cortical reorganization along key motor development scores

**Fig 5:**
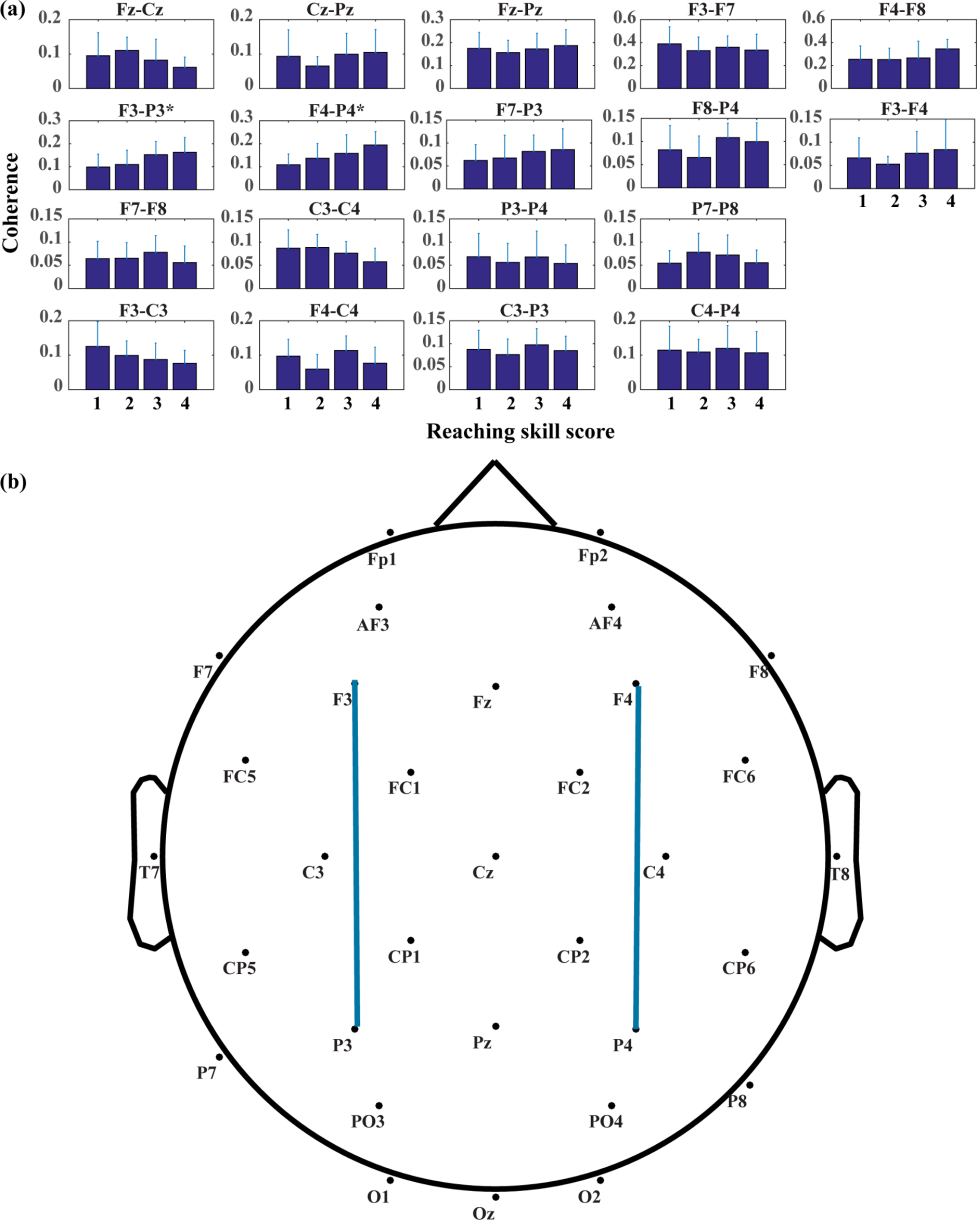
Changes in mean alpha coherence across different reaching skill scores. (A) Changing trends of mean alpha coherence for each electrode pair. In each subplot, bars present mean magnitudes of mean alpha coherence for sessions of the same reaching skill score, with corresponding standard deviation added on top. (B) Electrode pairs with significance in likelihood ratio tests. Electrode pairs with significance are connected with blue lines.

**Fig 6:**
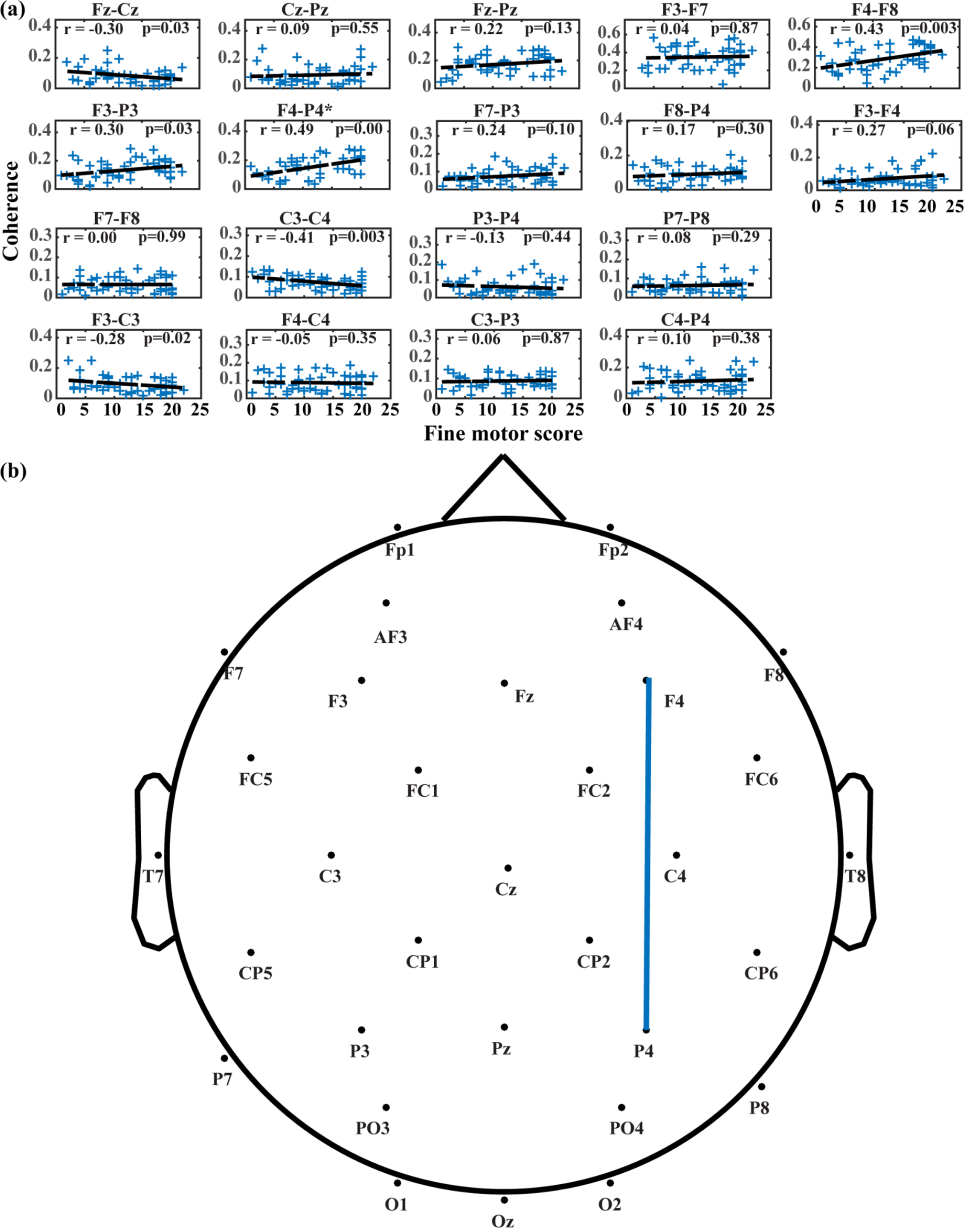
Changes in mean alpha coherence across fine motor scores from the Bayley test. (A) Changing trends of mean alpha coherence for each electrode pair. In each subplot, blue crosses denote magnitudes of mean alpha coherence for all sessions, and dashed lines are the least-squares fitted lines. (B) Electrode pairs with significance in likelihood ratio tests. Electrode pairs with significance are connected with blue lines.

**Fig 7:**
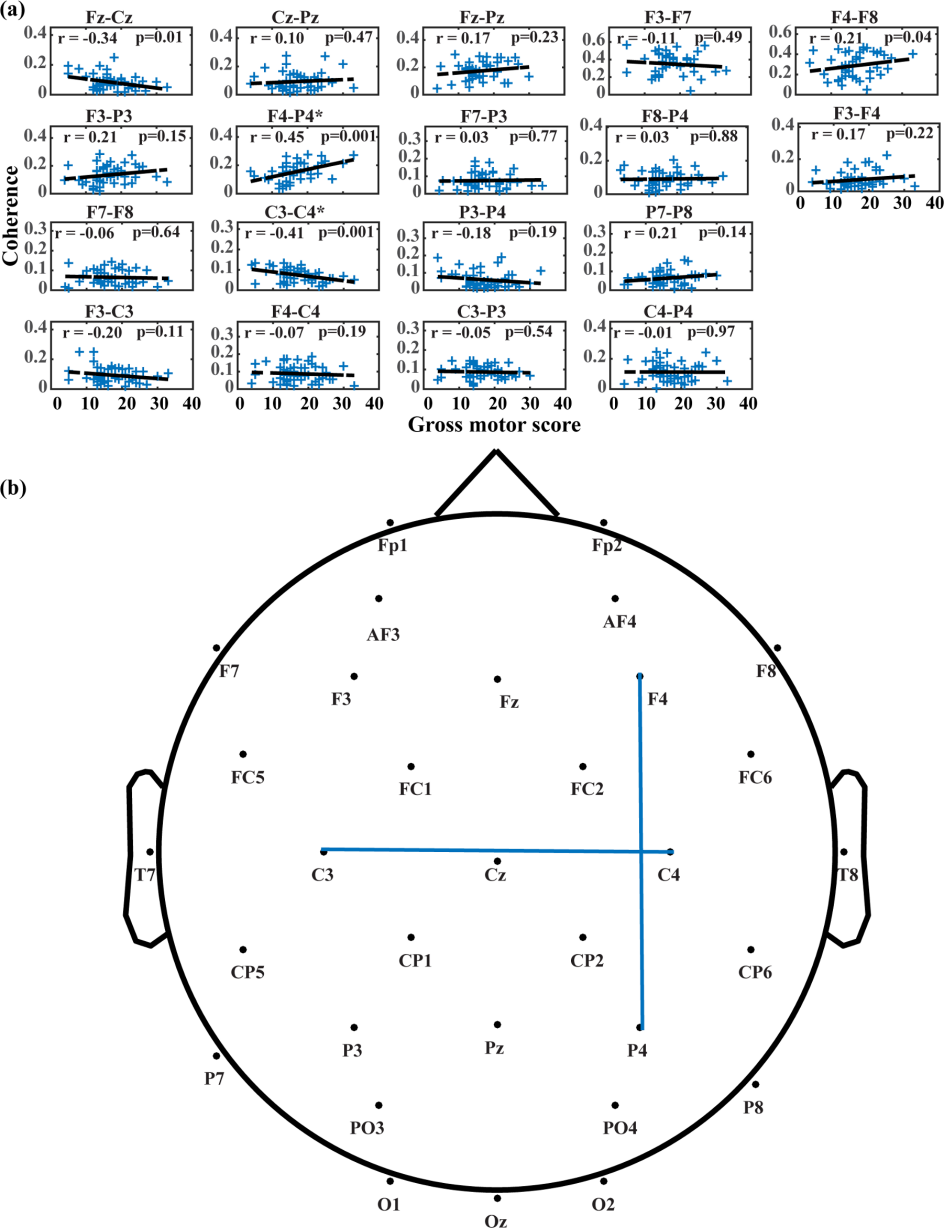
Changes in mean alpha coherence across gross motor scores from the Bayley test. (A) Changing trends of mean alpha coherence for each electrode pair. In each subplot, blue crosses denote magnitudes of mean alpha coherence for all sessions, and dashed lines are the least-squares fitted lines. (B) Electrode pairs with significance in likelihood ratio tests. Electrode pairs with significance are connected with blue lines.

Figs 5–7 present changes in connectivity among various brain regions across different motor development measurements acquired in present study. Fig 5 shows the connectivity changes with respect to reaching skill scores. 2 electrode pairs out of 18 present significant changes in connectivity, both associated with frontal-parietal network (F3-P3, χ^2^ = 7.509, *p < 0*.*0028*; F4-P4, χ^2^ = 10.769, *p < 0*.*0028*). The interhemispheric motor cortices (C3-C4) also present large variation with χ^2^ = 6.114 and *p = 0*.*006*. In these electrode pairs, bilateral frontal-parietal networks present increasing connectivity as reaching skill developing, while the interhemispheric connectivity in motor cortices presents a generally decreasing pattern. Fig 6 presents changes in mean alpha coherences across raw fine motor subscale scores from the Bayley test. Only right frontal-parietal network (F4-P4, χ^2^ = 11.349, *p < 0*.*0028*) demonstrates significant changes of connectivity across the specific motor development score. Other electrode pairs presenting large changes include left frontal-parietal network (F3-P3, χ^2^ = 4.319, *p < 0*.*05*), fronto-motor (Fz-Cz, χ^2^ = 5.803, *p < 0*.*05*), interhemispheric motor (C3-C4, χ^2^ = 5.803, *p < 0*.*05*), and right frontal and prefrontal (F4-F8, χ^2^ = 4.319, *p < 0*.*05*) cortices. As shown in Fig 6A, most of these electrode pairs show positive correlation with fine motor scores (r = 0.30 for F3-P3; r = 0.49 for F4-P4; r = 0.43 for F4-F8), while high negative correlation is revealed for interhemispheric motor cortices (r = -0.41 for C3-C4) and fronto-motor cortices (r = -0.30 for Fz-Cz).

Two electrode pairs are found to have significant connection changes related to raw gross motor subscale scores from the Bayley test, including right frontal and parietal (F4-P4, χ^2^ = 9.748, *p < 0*.*0028*), and left and right motor cortices (C3-C4, χ^2^ = 9.071, *p < 0*.*0028*) as shown in Fig 7. Similar to analysis with other motor developmental scores, positive correlations are uncovered for right frontal and parietal (r = 0.45), with negative correlation for interhemispheric motor regions (r = -0.41) and between frontal and motor cortices (r = -0.34).

A full list of likelihood ratio testing results testing for change in mean alpha coherence by chronological age, reaching skill, and Bayley fine and gross motor raw scores can be found in Table 1. Electrode pairs with significance from the tests are marked in bold and italic.

## Discussion

In this study, we showed the development of the peak in relative power in the 6–9 Hz frequency band of EEG. Previously, this peak has only been observed in older infants. It has been observed to be unstable at 5 months [12], but consistently observed by 7 [13] or 10 [12] months of age. We are the first to show that it is not present around 1 month of age and starts to appear across the following months (see Fig 1). The instability observed before 7 months of age is presumably due to known variability in rates of infant development, and further work is necessary to better understand cause and effect relationships. Furthermore, its spatial patterns and changing trend were explored (Fig 2). The results support that 6–9 Hz rhythm is a motor rhythm in infant EEG developing across the first half year of age, with the largest activation and age related changes at motor cortices.

In this study, we also began to explore whether changes in brain function were related to and/or reflective of the development of neuromotor control by exploring longitudinal changes in coherence within the 6–9 Hz range across different regions of the infant brain. Coherence generally increased in the bilateral frontal-parietal networks, while the interhemispheric connectivity in motor cortices generally decreased. When comparing likelihood ratio testing results testing for reaching skill, and Bayley fine and gross motor raw scores, there were significant impacts of all factors on change in mean alpha coherences in frontal-parietal, and interhemispheric motor cortex regions, supporting that changes in alpha coherence were related to changes in neuromotor skill. While age alone was shown to be a predictor of change in alpha coherence, the prediction could be more accurate when other factors evaluated in present study, including reaching skill and Bayley fine and gross motor raw scores, are also incorporated into the prediction model. This was a preliminary study with a small number of infants tested longitudinally at varying ages and reaching skill levels; we will use these results to design an adequately-powered study to develop norms and to investigate the relationships of interest identified here.

Some developmentalists believe that perceptual-motor connections are present at birth and their function is shaped by experience [14–16], while others suggest that both the connections and function are formed and shaped through critical sensorimotor experience [17,18]. Either way, the role of sensorimotor experience and learning are of crucial importance to the functional development of the infant. Being able to identify deviation from healthy brain function early in life would allow for early, targeted intervention to support optimal developmental outcomes.

### Importance of 6–9 Hz frequency in infant motor control

In adults, desynchronization in the 8–13 Hz range (also called the mu rhythm) is related to movement preparation while synchronization is related to movement inhibition [19–22]. In infants, however, it is the 6–9 Hz frequency range, as opposed to the 8–13 Hz range, that resembles the adult mu rhythm [12,23–25]. A case series showed that individual infants’ ability to hold up their head, sit, stand and walk was accompanied by increased power in the 7–10 Hz frequency band in the occipital region, however they did not explicitly test relationships between EEG and motor control [26].

Changes in the infant mu rhythm can be measured prior to the onset of crawling, between 5 and 7 months of age [2]. A study with 9-month old infants demonstrated alpha desynchronization prior to the initiation of an arm reach to grasp an offered toy [3]. While this study demonstrated that alpha desynchronization preceded intentional infant arm reaching movements in 9-month-old infants who were skilled reachers, it does not inform us about the development of reaching skill as they did not test younger, less skilled infants. The present study complements previous studies demonstrating a relationship between infant mu rhythm development and motor skills, while expanding upon them by assessing a novel group: younger infants who are developing reaching skill.

### Coherence as a measure of infant development

Coherence has been related to specific measures of infant cognitive development. An increase in coherence in 6–9 Hz range activity was observed between 5 and 10 months of age in a longitudinal study. The increase was related to changes in working memory processing as measured by the A-not-B task, with the interpretation being that frontoposterior functional connectivity is essential for developing executive abilities [27,28]. A cross-sectional study of 7 to 12-month-old infants demonstrated larger coherence of EEG signals in relation to their better performance on the A-not-B task. The changes in EEG were attributed to increased organization and excitability in the frontal region [13].

### Relationship between coherence and motor development

Researchers have measured differences in the coherence of resting EEG signals of 8-month-old infants with various amounts of crawling experience. Higher coherence in early crawlers compared to infants who were not yet crawling was attributed to increased cortico-cortical connections emerging due to the new experience, while lower coherence in experienced crawlers was attributed to pruning of synapses with further experience [4]. Another study found significant differences in coherence among 12-month-old infants with varied levels of walking experience. The authors interpretation was that as infants acquire walking experience and decouple their arms, coherence decreases as overabundant synapses are pruned due to increased regional differentiation [5]. In the present study, two major changes in connection are revealed consistently along different developmental factors. The increase in coherence between frontal and parietal cortices indicates strengthening fronto-parietal network along maturation and motor skill development. This may implicate adaptation of brain connection towards improved ability in motor planning and visuospatial attention related to motor skill development. Another major change takes place between left and right motor cortices, presenting a decreasing pattern. Such interhemispheric desynchronization is consistent with lateralized control scheme of motor functions in motor cortex. Hence, the decrease in interhemispheric motor connection could be a biomarker of lateral specification of motor control. However, no metric for lateralization of motor control is available in present study. Further effort is needed to validate this relation in future studies.

### Limitations and conclusions

This was a preliminary study with a small number of infants tested longitudinally at varying ages and reaching skill levels. We only assessed motor development in this analysis, however infants were also advancing in the area of cognitive and language development. In addition, there are other potential EEG measures of interest, such as source localization.

In regard to EEG referencing, we chose to use cortical reference points for EEG processing, similar to most of previous infant motor studies. We chose this approach as it allows us to directly compare and contrast our results with previous work studying older infants. This is of key importance as understanding the developmental trajectory is our goal. There has been much recent discussion, however, as to selection of the EEG reference as “a fundamental technical issue that has yet to be resolved…. Since EEG amplifiers measure potential difference between the activities recorded by two electrodes, in addition to the active electrode, one must employ a reference electrode which should ideally be at zero….Examples of such references are the unilateral-mastoid, ear, linked mastoids or ears, vertex, the tip of the nose, neck ring, etc. Unfortunately all such references are doomed to fail since there is no point on the scalp or body surface where the potential is actually zero or a constant [29].” An alternative approach is the reference electrode standardization technique (REST). REST standardizes a reference point at infinity in recovering temporal information in EEG [30, 31]. As more and more studies show the choice of reference points impacts EEG spectral and connectivity analysis [32–35], the generalizability of our results is limited. In the future, we would like to compare and contrast the infinity reference point method as it might lead to additional insight about infant motor development. In order to do this, we will need to re-do all analyses using EEG data with a different reference (REST), followed by filtering, segmenting, cleaning, and analyzing the data in the same way we did here. Once that is achieved, we can compare and contrast the REST reference findings with our original findings in a methodological study. To summarize, the choice of an EEG reference has a fundamental impact on the results that are obtained. While our choice of reference here allows us to place our findings in the context of the existing infant EEG motor development literature, newer approaches may be more accurate and need to be explored.

We will use the results of the current study to design adequately-powered studies 1) to develop norms for typical development that may aid in the early detection of atypical brain development and/or be used as an objective measure for monitoring developmental progress or outcomes related to intervention 2) to investigate the relationships of interest identified (lateralization of motor control, motor development in relation to language and cognitive development).
